# Possible Biases of Researchers’ Attitudes Toward Video Games: Publication Trends Analysis of the Medical Literature (1980–2013)

**DOI:** 10.2196/jmir.5935

**Published:** 2016-07-18

**Authors:** Aviv Segev, Mitchell Rovner, David Ian Appel, Aaron W Abrams, Michal Rotem, Yuval Bloch

**Affiliations:** ^1^ Shalvata Mental Health Center Hod Hasharon Israel; ^2^ Sackler School of Medicine Tel Aviv University Tel Aviv Israel; ^3^ Albert Einstein College of Medicine/Montefiore Medical Center Bronx, NY United States; ^4^ University of Vermont Medical Center Burlington, VT United States; ^5^ SUNY Downstate Medical Center Brooklyn, NY United States

**Keywords:** video games, publication trends, bias

## Abstract

**Background:**

The study of video games is expanding, and so is the debate regarding their possible positive and deleterious effects. As controversies continue, several researchers have expressed their concerns about substantial biases existing in the field, which might lead to the creation of a skewed picture, both in the professional and in the lay literature. However, no study has tried to examine this issue quantitatively.

**Objective:**

The objective of our study was to examine possible systematic biases in the literature, by analyzing the publication trends of the medical and life sciences literature regarding video games.

**Methods:**

We performed a complete and systematic PubMed search up to December 31, 2013. We assessed all 1927 articles deemed relevant for their attitude toward video games according to the focus, hypothesis, and authors’ interpretation of the study results, using a 3-category outcome (positive, negative, and neutral). We assessed the prevalence of different attitudes for possible association with year of publication, location of researchers, academic discipline, methodological research, and centrality of the publishing journals.

**Results:**

The attitude toward video games presented in publications varied by year of publication, location, academic discipline, and methodological research applied (*P*<.001 for all). Moreover, representation of different attitudes differed according to centrality of the journals, as measured by their impact factor (*P*<.001).

**Conclusions:**

The results suggest that context, whether scientific or social, is related to researchers’ attitudes toward video games. Readers, both lay and professional, should weigh these contextual variables when interpreting studies’ results, in light of the possible bias they carry. The results also support a need for a more balanced, open-minded approach toward video games, as it is likely that this complex phenomenon carries novel opportunities as well as new hazards.

## Introduction

Playing video games is a worldwide, significant social phenomenon with possible effects on life and health. Two main attitudes, often polarized, have dominated the interpretation of the consequences of playing video games on well-being since the early days of research in this field [1], with this heated debate continuing today [2]. On one hand, there are those who emphasize the advantages of video games, including beneficial uses of the media [3] such as cognitive enhancement [4,5], rehabilitation [6,7], and prosocial behavior [8]. On the other hand, studies have reported harmful effects of video games on players, including academic deterioration [9], attention and psychosocial problems [10-12], violent behavior [13], and further deleterious effects.

Many possible applications of video games, either commercial or goal-oriented (“serious games”), in the fields of health and medicine were studied in the literature: promoting health behaviors [14,15], motor skills and balance [16,17], cognitive rehabilitation [6,18], medical training [19,20], and even psychotherapy [21,22]. As the literature indicates the possible negative outcomes of video games, and recurrent warnings are being published by leading medical authorities [23], clinicians and policy makers face a complex challenge: to translate the possibilities and applications of video games into clinical practice and official statements, in light of the confusing and contradictory evidence.

These dichotomous views of video games may drive professionals to choose a dichotomous stance, either positive or negative, on video games. These stances have a major impact, especially among physicians, as they may be transmitted, in turn, to their patients and students.

As video game variety, usage, content, and context are widely diverse and have become a part of modern life, Bavelier and Green stated that “One can no more say what the effects of video games are, than one can say what the effects of food are,” implying that “the devil is in the details” [2].

Ideally, we look for science to bring forth results and data that will reveal the costs and benefits of this practically universal behavior. However, this optimistic view overlooks the fact that research only answers the hypothesis suggested. Thus, when studying new, emotion-provoking phenomena, the biases of researchers, on which the basic hypotheses are based, might affect and skew the focus of research and the interpretation of its results.

Such biases have been suggested in the literature concerning video games, which might cause a disconnect between the studies’ findings and their interpretations in the public and professional literatures [24-26]. Along this line of thought, familiarity with computer games has been identified as a possible moderator of one’s beliefs about computer games [27].

In this study, we set forth to examine trends and possible bias in the medical literature focusing on video games, by examining trends by time of publication, country of origin, medical discipline, and research methodology. Revealing such trends may raise awareness of researcher bias, thus helping to formulate a clearer understanding of the interpretation of studies evaluating the risks and rewards of video games. We set forth to examine such biases by examining the researchers’ attitudes reflected in the study publication. By attitude, we mean “A settled way of thinking or feeling about something” [28].

## Methods

### Search Strategy

We conducted a systematic search on the PubMed database for all articles published up to December 31, 2013, using synonyms for video games (plural): videogames, video games, “video games”[MeSH] (major and subtypes), electronic games, and “computer games.”

### Database Assembly and Variables of Interest

We classified all results according to the following parameters: year of publication, publishing journal, and country of origin (based on the affiliations of the first author). Using the ISI Web of Knowledge (now the Web of Science), we added subject categories to each record, as well as the impact factor of the publishing journal. We then manually accessed each article and read all possible abstracts. If an abstract was not available, or not coherent, we accessed and read the article itself. That process allowed 3 fields to be added to each record: (1) relevance of the article was assessed (relevant/not relevant), based on the role computer games had in the study, because articles may describe video gamers as a control group or as the placebo task for a cognitive test, (2) article type was determined (eg, case study, expert opinion, cross-sectional study, randomized controlled trial), and (3) attitude was assessed on a 3-category variable (as either positive, neutral, or negative), based on the focus, hypothesis, and the article authors’ interpretation of the study results and conclusions. Studies hypothesizing that video games increase aggressiveness would be considered to have a negative attitude, but negative results in such a study and a conclusion encouraging doubts about the concept of video game-induced aggressiveness would be considered neutral. On the other hand, a study examining the contribution of active video games to balance rehabilitation would be classified as positive. Similarly, a study examining this issue and reporting a negative result and urging caution when implementing video games in balance rehabilitation would also be classified as neutral.

### Data Quality Assurance

We divided the process of determining the attitude of the article and ensuring interrater reliability into several steps. Initially, 4 classifiers (MR, DIA, AWA, MR) classified 150 articles, each of which was also examined by the lead researcher (AS); all of the researchers discussed conceptual questions. Next in the classification, each of the classifiers worked alone, while every question that arose was discussed with the lead researcher. If considerations about the classification process seemed to have generalization potential, the discussion was relayed to the other classifiers.

After finishing the classification, and to ascertain reliability, a different classifier, who was blinded to the previous categorization, reanalyzed 10% of the sample, randomly selected by computer. These decisions were compared in order to examine agreement.

### Statistical Analysis

Statistical analysis was performed using IBM SPSS version 16 software (IBM Corp). First, we examined each variable of interest. Spearman correlation examined trends over time. For categorical variables (researchers’ location, medical discipline, research methodology, and impact factor), we used chi-square analysis with a follow-up post hoc 2×3 chi-square for each of the subcategories of variables.

## Results

We retrieved 3223 articles in total, of which 170 were duplicates, leaving 3053 articles. For 230 of those (7.53%), we gathered the information from the article itself, as the article had no abstract, or we deemed the abstract not to be informative enough for classification. Only 75 articles (2.46%) had no abstract and the article itself was unattainable, leaving only the title, affiliations, and PubMed’s medical subject headings (MeSH) to rely on for classification. In those cases, if the information was not sufficient, we excluded the article from the analysis. For all other articles (n=2748, 90.01%), we based classification on the abstract. We deemed 1126 articles to be irrelevant, as video games were not the focus of the article, leaving 1927 articles, published in 300 different journals, from 64 different countries.

Blinded agreement between researchers on the article’s attitude was substantial (κ=.77, *P<*.001). Furthermore, virtually all of the disagreements were either positive versus neutral or negative versus neutral. Only 1 study of the 186 in the verification process was assigned a contrasting attitude (negative-positive); thus, agreement regarding attitude direction was almost perfect (κ=.99, *P<*.001).

Overall, we classified 812 of the 1927 (42.14%) as negative-attitude publications, 301 (15.62%) as neutral, and 814 (42.24) as positive-attitude publications.

### Change in Publication Number and Attitudes Over Time

**Figure 1:**
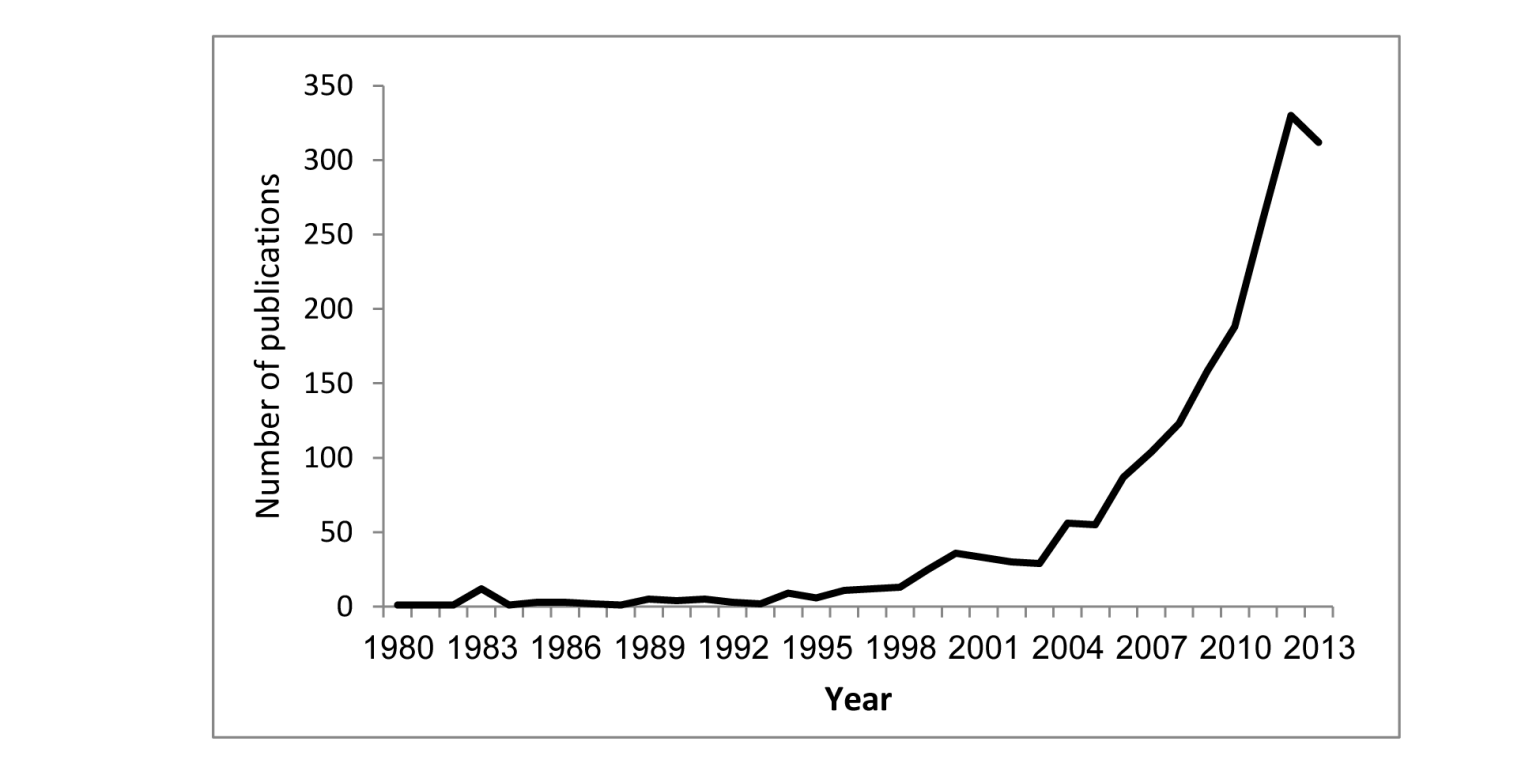
Number of video game-related publications, 1980–2013.

We found a significant and meaningful Spearman correlation between the number of publications and the year (*r*=.946, *P<*.001), from the first and only publication in 1980, to 312 articles in 2013 (Figure 1).

**Figure 2:**
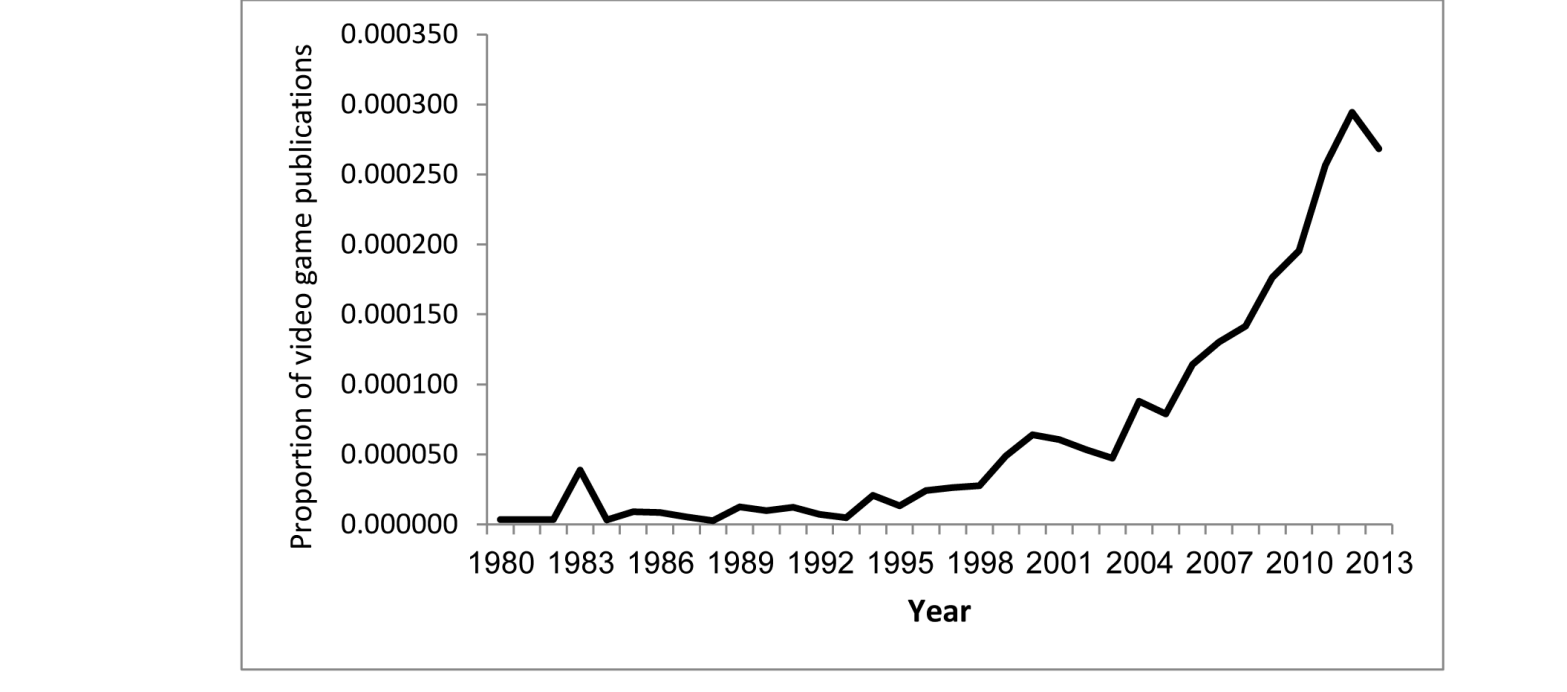
Proportion of video game-related publications, 1980–2013.

Furthermore, the proportion of video game publications was also positively correlated with the year (*r*=.927, *P<*.001), from 1 out of 279,486 (0.00049%) in 1980, to 312 out of 1,136,703 (0.027%) in 2013, for growth by a factor of 55.1 (Figure 2).

**Figure 3:**
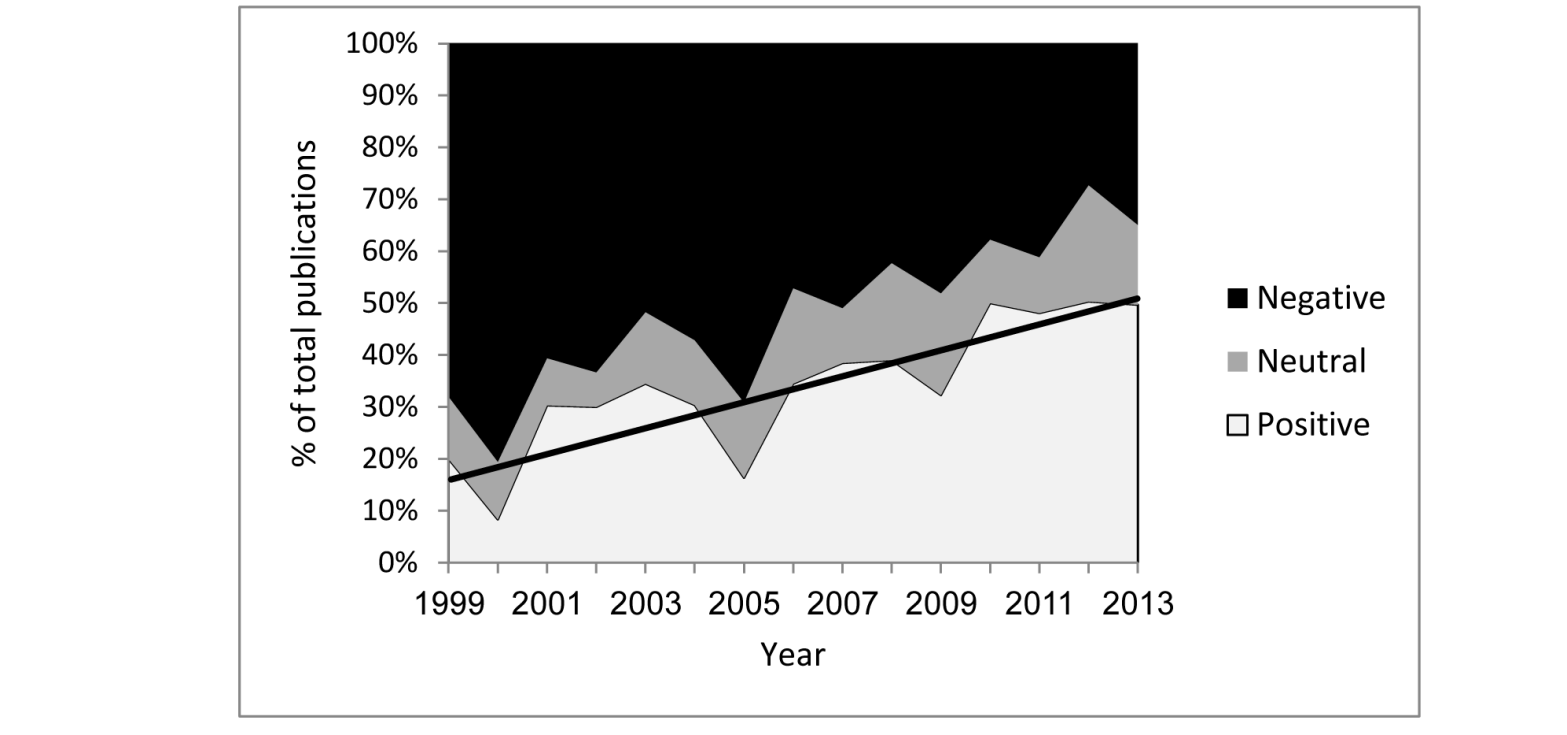
Proportions of attitudes toward video games over time, 1999–2013. The line demonstrates the positive linear trend of positive-attitude publications.

As the number of publications per year was very low until 1999 (<15 per year, for a total of 101 articles in 19 years), we excluded these years from this specific analysis only, as even a single article would create a major shift in the said year. As the years progressed, the proportion of negative publications dropped (*r*=–.907, *P<*.001), while the number of positive and neutral publications increased (*r*=.87, *P<*.001 and *r*=.519, *P=*.047, respectively) (Figure 3).

As we excluded the first 19 years of video game studies due to the scarcity of articles each year, we analyzed those years in 2 large fragments: 1980–1989 and 1990–1998.

Over the first 10 years of research, 34 articles were published, 19 (56%) of them with positive attitude, and 11 (32%) with negative attitude. Over the next 9 years (1990-1998), an additional 67 articles were published, 25 (37%) of them with positive attitude and 32 (48%) with negative attitude.

### Change in Attitudes Across Regions

As 64 different countries contributed to the pool of articles dealing with video games, we grouped countries according to location (United States, Canada, Europe, Eastern Europe, Australia and New Zealand, Middle East, Asia, Africa, and South America). We combined groups of countries that contributed fewer than 100 articles into 1 group, leaving 6 regions: Australia, Canada, Europe, Asia, United States, and other. A chi-square test revealed a significant association between attitude distribution (positive, negative, or neutral) and the region from which the article originated (*P<*.001). A post hoc test of 2×3 chi-square revealed a significant increase in positive articles (with a reciprocal decrease in negative articles) in the United States (*P<*.001) and Canada (*P=*.045) and a decrease in positive articles (with a reciprocal increase in negative and neutral articles) in Asia (*P<*.001) (Table 1). In Europe there was a trend to increased negative articles and decreased positive articles (*P=*.05).

**Table 1:** Changes in attitudes toward video games across regions, 1980–2013, as reflected in the change in proportion of video game-related articles.

Region	Countries	Total no. of articles	Change in attitude (%)			*P* value
			Negative attitude	Neutral attitude	Positive attitude	
US	United States	804	–5.7%	–0.8%	+6.6	*<*.001^a^
Canada	Canada	119	–9.3%	–1.3%	+10.7%	.045
Australia	Australia, New Zealand	103	+4.5%	–3.0%	–1.4%	*>*.1
Europe	Austria, Belgium, Denmark, Finland, France, Germany, Iceland, Italy, Ireland, Luxembourg, the Netherlands, Norway, Portugal, Scotland, Spain, Sweden, Switzerland, United Kingdom	554	+3.9%	+0.3%	–4.1%	.05
Asia	Bangladesh, China, Hong Kong, India, Indonesia, Japan, Korea, Malaysia, Philippines, Singapore, South Korea, Taiwan, Thailand, Vietnam	176	+11.9%	+6.0%	–17.8%	*<*.001^a^
Other	Eastern Europe (Bosnia-Herzegovina, Croatia, Czech Republic, Hungary, Lithuania, Poland, Romania, Russia, Serbia, Ukraine); Middle East (Egypt, Greece, Iran, Israel, Lebanon, Oman, Saudi Arabia, Turkey); South America (Argentina, Brazil, Chile, Colombia, Jamaica, Mexico, Puerto Rico); others (Georgia, Nigeria, South Africa, mixed countries)	171	+5.9%	–0.4%	–5.4%	*>*.1

^a^Significance remains after Bonferroni correction.

### Change in Attitudes Across Disciplines

Using subject categories, as defined by the Journal Citation Reports (Thomson Reuters, New York, NY), we classified articles by discipline according to the journal in which they were published. Thus, we classified a portion of the 1927 articles in more than 1 domain: 1352 publications (70.16%) were classified to 1 discipline, 490 (25.43%) to 2 disciplines, 38 (1.97%) to 3 disciplines, and 4 (0.21%) to 4 disciplines. We did not assign 43 publications (2.23%) to any discipline, as the publishing journals were not listed in ISI and we could not unequivocally derive the journal discipline from the name of the journal.

The 8 defined disciplines were pediatrics, psychiatry and psychology, neurology, basic sciences, nonmedical and technology, public health and environment, rehabilitation, and internal and general medicine (comprising several fields in medicine in which the number of publications was low, such as ophthalmology, nursing, and family practice). Other than general and internal medicine, the most prominent field was psychiatry and psychology, with 572 (29.68%) of all publications in the field of video games. The next most prominent field was pediatrics (326, 16.92%), followed by public health and environment (311, 16.14%).

A chi-square test revealed a significant association between attitude distribution and disciplines (*P<*.001).

A post hoc test of 2×3 chi-square revealed a reduced number of positive articles (with a reciprocal increase in neutral articles) in psychiatry and psychology (*P<*.001), an increased number of positive articles (with a reciprocal decrease in neutral articles) in general and internal medicine (*P=*.001), an increased number of positive articles (with a reciprocal decrease in neutral and negative articles) both in rehabilitation and in nonmedical and technology domains (*P<*.001 for both), an increased number of negative articles (with a reciprocal decrease in positive articles) in pediatrics (*P<*.001), and an increased number of negative articles (with a reciprocal decrease in neutral articles) in public health and environment (*P<*.001). No correlation was found for neurology and basic sciences (Table 2).

**Table 2:** Change in attitudes toward video games across different disciplines, 1980–2013, as reflected in the change in proportion of video game-related articles.

Subject	Total no. of articles	Change in attitude (%)			*P* value
		Negative attitude	Neutral attitude	Positive attitude	
Psychiatry and psychology	572	+1.1%	+13.9%	–14.9%	*<*.001^a^
Pediatrics	326	+17.4%	–1.2%	–16.1%	*<*.001^a^
Rehabilitation	135	–39.1%	–10.4%	+49.7%	*<*.001^a^
Nonmedical and technology	206	–29.0%	–6.4%	+35.5%	*<*.001^a^
Neurology	161	–5.5%	+1.8%	+3.8%	*>*.1
Basic sciences	93	–3.4%	–0.5%	+4.0%	*>*.1
Public health and environment	311	+8.7%	–5.3%	–3.3 %	.001^a^
General and internal medicine	658	–1.4%	–5.6%	+7.0%	*<*.001^a^

^a^Significance remains after Bonferroni correction.

### Change in Attitudes Across Methodological Approaches of Studies

We then divided the articles into 3 categories based on study design: observational studies, interventional studies, and study aggregations (reviews and meta-analyses).

As methodological requirements and evidence-based approaches have shifted greatly since 1980, we examined the correlation between study methodology and the year of publication.

We found a significant and meaningful Spearman correlation between the methodological approach and the year: as the years progressed, the proportion of observational studies declined (*r*=–.75, *P=*.001) and that of interventional studies increased (*r*=.63, *P=*.01). No correlation was found with the number of aggregation-based articles (*r*=–.086, *P>*.1). We then examined whether there was an association between the research methodology and attitude. The correlation was significant (*P<*.001): positive articles increased in interventional studies (*P<*.001), with a reciprocal decline in negative articles, while negative articles increased in observational studies, with a reciprocal decline in positive articles (Table 3).

**Table 3:** Change in attitudes toward video games across methodological approaches, 1980–2013, as reflected in the change in proportion of video game-related articles.

Type of study	Total no. of articles	Change in attitude (%)			*P* value
		Negative attitude	Neutral attitude	Positive attitude	
Observational	1081	+16.0%	+1.1%	–16.9%	*<*.001^a^
Interventional	686	–25.8%	–0.9%	+26.8%	*<*.001^a^
Aggregated	160	+2.9%	–3.1%	+0.3%	*>*.1

^a^Significance remains after Bonferroni correction.

### Change in Attitude Across Journal Centrality

Based on the impact factor (defined by Journal Citation Reports), we divided the journals into 3 groups: lower impact factor (ranging from 0 to 2.0 or nonlisted), medium impact factor (ranging from 2.001 to 4.0), and high (≥4.001). The groups comprised, respectively, 908 (47.12%), 668 (34.67%), and 351 (18.21%) of the 1927 publications. A chi-square test revealed a significant association between the impact factor group and attitude of the articles (*P<*.001). The low impact factor group tended to publish more positive articles (with a reciprocal decrease in neutral and negative articles) (*P<*.001), whereas both the medium and high impact factor groups tended to publish fewer positive articles, with a reciprocal increase in neutral articles in the medium group (*P<*.001) and a reciprocal increase in negative articles in the high impact factor group (*P=*.008) (Table 4).

**Table 4:** Change in attitudes toward video games across journal centrality, 1980–2013, as reflected in the change in proportion of video game-related articles.

Impact factor group	Total no. of articles	Change in attitude (%)			*P* value
		Negative attitude	Neutral attitude	Positive attitude	
Low (0–2.0)	908	–3.1%	–4.1%	+7.4%	*<*.001^a^
Medium (2.001–4.0)	668	+0.4%	+6.6%	–6.9%	*<*.001^a^
High (≥4.001)	351	+7.5%	–1.6%	–5.7%	.008^a^

^a^ Significance remains after Bonferroni correction.

## Discussion

### Principal Findings

The number of articles reporting studies of video games is increasing rapidly. It seems that the attitude toward video games is affected by the year of publication, the region of origin of the lead researcher, the discipline from which the article stems, and the research method applied. Moreover, it seems that the representation of different attitudes varies according to the centrality of the journal, as measured by its impact factor.

Surprisingly, in the early years of video games research, while opinion leaders were speaking against video games and their deleterious effects [1], most articles presented a positive attitude. The positive trend prevailing in the 1980s was reversed during the 1990s, when negative attitudes toward video games were reflected in nearly half of the publications. A possible explanation for the proliferation of positive articles in the 1980s is the novelty of this subject, with enthusiastic researchers focusing on this new field and its opportunities. When viewed by year of publication, positive attitudes increased over time (excluding the early “pioneer” years). One possible explanation is that attitudes toward video games are affected by prior experience with the field, and that gaining experience with the medium, and integrating younger researchers who have been exposed to video games all their lives, would lead to a more positive approach. Another possibility would be to view the attitude of the medical research community in a similar way to the patterns of technology adoption. The temporal curves, delineating a rise of positive articles in the 1980s, a strong shift toward negative articles in the 1990s, and then a gradual incline of positive attitudes, follows the trends depicted in Gartner’s hype cycles portraying the adoption of new technologies [29].

The assessment based on the country of origin supports the “acquaintance” hypothesis [27]: articles from the United States, a leader in the video game industry, tended to be positive. Articles from Asia, which comprises a mesh of traditional and modern cultures, and with exponential growth of technological penetration and video games, leaned toward negative attitudes. This is possibly a reflection of the repercussions of a rapidly changing culture and assimilation of changes in lifestyle.

Among the different medical disciplines, most of the findings can seem trivial. One could assume that basic science, by its very nature, would tend to lack a polarized attitude. Also, it is not surprising that the field of rehabilitation, which seeks a measure of improvement, would be positively biased. The same bias would be very plausible in the technological disciplines. Public health, though, a discipline that tends to look for risk factors and prevention measures, would understandably be biased toward the more negative attitudes. Neurology seems to be balanced. This leaves 2 disciplines with an intriguing tendency toward the negative: psychiatry and psychology, and, even more so, pediatrics. One can hypothesize that the rapid dissemination of and increase in the number of video games used in the field of pediatrics, along with a more protective and more pronounced generation gap, could be a possible explanation. Another possible explanation would be a specific “toxic” effect specific to early development and mental health. This view is in accord with when video games are being considered as a type of behavioral addiction. However, the positive-attitude studies published in the educational field, as well as in neurology and cognitive rehabilitation, strengthen the suspicion of a negative-attitude bias both in pediatrics and in psychiatry and psychology research.

The methodological partition results may not be surprising. Observational studies are often directed toward negative outcomes (eg, risk factor), while interventional studies usually seek benefits (although difficult to examine systematically, it seems that it is less common for a study to manipulate an intervention that will intentionally cause negative effects than to intervene in order to achieve a more favorable outcome).

The results of this study suggest a possible publication bias as a factor in the basic attitude of the article: negative-attitude articles are more likely to get published in a high impact factor journal. When studying a common phenomenon, such as video games, observational studies (which, as suggested, may favor a negative attitude) offer access to large populations, thus enabling stronger methodology. This might explain the bias of the higher impact factor journals toward publishing studies with a negative attitude. One can expect that, in the coming years, as the technological possibilities of interventional studies improve and as the mass of interventional studies increases, more methodologically robust interventional studies will find their way to more influential journals.

### Limitation

Though the study covered the entire literature indexed in PubMed, our scope was limited to medical and life sciences-related publications. As such, we cannot attest to attitudes in other academic branches dealing with video games, such as education or communications. However, as few such articles have been indexed in PubMed, and consequently analyzed in this study, the general approach toward computer games seems to be more positive. It should be noted that, as the study focused on attitudes in the medical literature, we searched only the PubMed database. Furthermore, we might have captured additional studies dealing with video games by using different keywords in our search strategy.

An additional limitation that should be noted is that we coded attitudes manually, according to human judgment, which is vulnerable to mistakes and disagreements. However, as the blinded agreement between the authors was very high, it is not likely that misclassification of articles interfered with the results. We chose to focus in this study on video games rather than social media or the internet as a whole, as the topic of video games is grounds for even greater disagreement. As internet use and email can be considered an essential part of the normal modern world (eg, for work, in the household, and for academic assignments), video games are considered “avoidable” and not a necessity, and thus their costs and benefits should be studied more carefully. Although we chose to focus on video games, in reality, the line between social media and video games has become blurred, because, unlike in the past [30], video games now comprise extensive social media and multiplayer options [31], a problem that has been raised in the debate surrounding the new definition of internet gaming disorder in the fifth edition of the *Diagnostic and Statistical Manual of Mental Disorders* [32,33].

### Conclusions

As suggested in the literature [24-26], biases do seem to exist, and recognizing these biases is important for the scientific community studying video games. It allows the reader to put a new study into a wider context, which seems to play a major role, according to our study, and thus could provide a better perspective when interpreting information. Furthermore, these biases should serve as a wake-up call and remind us to keep an open mind about this phenomenon, carrying (as all new phenomena do) both positive and negative perspectives, which are probably intertwined with one another more often than not. Further research should examine possible biases within specific subjects, particularly subjects that are fiercely debated, such as violence, addiction, and physical health implications.

## Abbreviations

MeSH: Medical Subject Headings
